# A patient with multiple synchronous gliomas of distinctly different grades and correlative radiographic findings

**DOI:** 10.4103/2152-7806.69375

**Published:** 2010-09-16

**Authors:** Fadi Nakhl, Edwin M. Chang, John S.C. Shiau, Anthony Alastra, Monika Wrzolek, Marcel Odaimi, Mark Raden, Jamie E. Juliano

**Affiliations:** Department of Hematology/Oncology, Staten Island University Hospital, Neuroscience Associates of New York, New York; 1 Division of Neurosurgery, Staten Island University Hospital, Neuroscience Associates of New York, New York; 2 Department of Pathology, Staten Island University Hospital, Neuroscience Associates of New York, New York; 3 Department of Radiology, Staten Island University Hospital, Neuroscience Associates of New York, New York

**Keywords:** High-grade astrocytoma, glioblastoma multiforme, low-grade astrocytoma, magnetic resonance imaging, multicentric, multifocal

## Abstract

**Background:**

Multiple gliomas represent approximately 2 to 5% of all high-grade gliomas which are categorized as multifocal or multicentric depending on the timing, location and pattern of spread. We present a patient with bi-hemispheric, noncontiguous, low- and high-grade gliomas proven by biopsy. She underwent surgical excision and radiotherapy, but unfortunately succumbed to her disease shortly thereafter.

**Case Description:**

A 64-year-old female presented to the hospital with confusion, disorientation and retrograde amnesia after an unwitnessed fall. There were no symptoms of headaches or visual disturbances before presentation. Magnetic resonance imaging (MRI) with and without gadolinium revealed a nonenhancing left temporal lobe mass without surrounding edema, an enhancing left frontal lobe mass with surrounding edema, and an enhancing right parietal lobe mass with surrounding edema. The patient underwent a left frontal craniotomy with gross total resection of the left frontal mass and a left temporal craniotomy, anterior temporal lobectomy and sub-total resection of the temporal lobe mass. Intraoperative Brainlab® image-guided navigation was used. Postoperative treatment consisted of radiotherapy.

**Conclusion:**

This is the first reported case of multiple separate glial tumors, each with differing grades in which an MRI can be correlated with the tissue diagnoses. This case also highlights the possible mechanisms of transformation of glial tumors in the continuum from benign to malignant forms, lending insight to the possibility of using advanced genetic analysis in the treatment and diagnosis of these entities.

## INTRODUCTION

Glial tumors represent 42% of all primary adult CNS neoplasms and over 75% of them are malignant high-grade gliomas. Glioblastoma multiforme, the most common malignant glioma, is a tumor with a high rate of proliferation and recurrence. It can present as multiple synchronous lesions due to its aggressive biological behavior.[18] Multiple gliomas are a well-recognized but relatively uncommon entity, with a reported incidence of approximately 2 to 5% of total high-grade gliomas.[2,11] They are often designated as multifocal or multicentric lesions. Multifocal gliomas are the result of dissemination of glioma cells from a primary focus to other parenchymal areas via the cerebrospinal fluid, meninges or white matter tracts. Multicentric gliomas are tumors arising independently in more than one site of the brain with absence of seeding along easily accessible routes.[2,5,14] Multiple gliomas may be classified depending on the time of presentation as synchronous if the tumors are detected on initial examination, or metachronous if detected during treatment.[19] Low-grade gliomas are relatively slow growing tumors accounting for 15 to 35% of all CNS tumors. They are classified as grade I or II by the World Health Organization (WHO) grading system [Table 1]. This category includes virtually every tumor of glial origin that is not overtly malignant at the time of initial diagnosis. Nonetheless, grade II gliomas can invade adjacent normal tissue and may have a low rate of recurrence after total resection. They represent a remarkable diversity of lesions characterized by various histological features that have different outcomes in term of differentiation and transformation to malignant gliomas. The major cause of mortality for patients with low-grade astrocytoma is progression to high-grade astrocytoma.[15]

**Table 1:** World health organization designation and classification of gliomas

Grade	Designation	Classification
I	Pilocytic astrocytoma	Glial tumors with little cellularity and minimal pleomorphic changes
II	Astrocytoma (low grade, diffuse, infiltrative, fibrillary)	Increased cellularity and atypia, without mitosis, endothelial proliferation or necrosis
III	Anaplastic astrocytoma (malignant)	Exhibits mitosis but no endothelial proliferation or necrosis
IV	Glioblastoma multiforme (malignant)	Highly cellular, nuclear and cellular pleomorphism, endothelial proliferation, high mitotic activity, and often, necrosis

## CASE REPORT

A 64-year-old female with an unremarkable past medical history was found unconscious after sustaining an unwitnessed fall resulting in an abrasion to her forehead.

She was confused, disoriented, and did not recall the circumstances of the event. Retrospectively, a seizure may have been the cause of the fall. The patient denied headaches or visual disturbances before presentation. On neurological physical exam the patient was disoriented, but had no apparent focal neurologic deficit. Her gait was steady and the rest of her physical exam was unremarkable.

MRI of the brain with and without gadolinium was performed. Three lesions were detected. A left temporal lobe lesion was identified and was slightly hypo-intense on T1-weighted imaging and hyper-intense on T2-weighted imaging with no associated gadolinium enhancement or surrounding edema [Figure 1a; 2a,b]. The left frontal lobe lesion was predominantly hypo-intense on T1 and hyper-intense on T2-weighted images. It enhanced in an irregular ring-like fashion with surrounding edema [Figure 1b]. In the right parietal lobe there was an enhancing lesion predominantly hypo-intense on T1 and hyper-intense on T2-weighted images [Figure 1c]. A metastatic work-up consisting of bone scan and CT scans of the chest, abdomen and pelvis were negative.

**Figure 1a:**
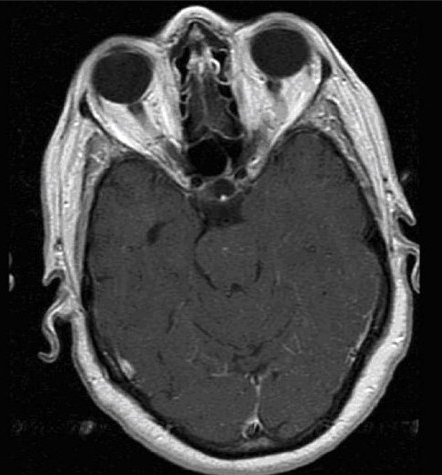
(a)T1-weighted MR images with gadolinium. Left temporal lobe tumor shows no enhancement but mild mass effect on the left ambient cistern

**Figure 1b:**
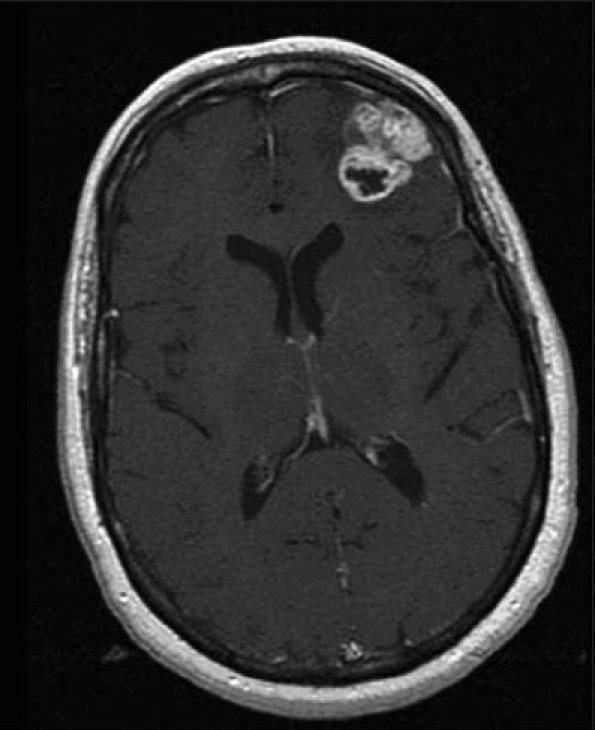
T1-weighted MR images with gadolinium. Nonhomogeneously enhancing left frontal tumor

**Figure 1c:**
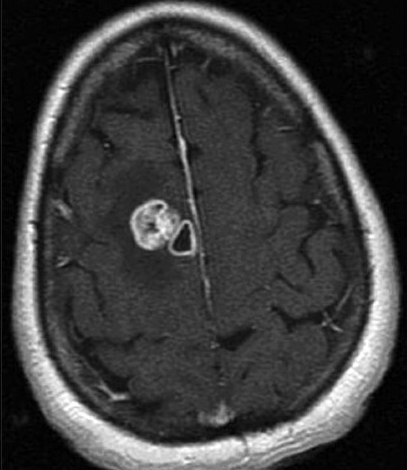
T1-weighted MR images with gadolinium. Nonhomogeneously enhancing right parietal tumor

**Figure 2a:**
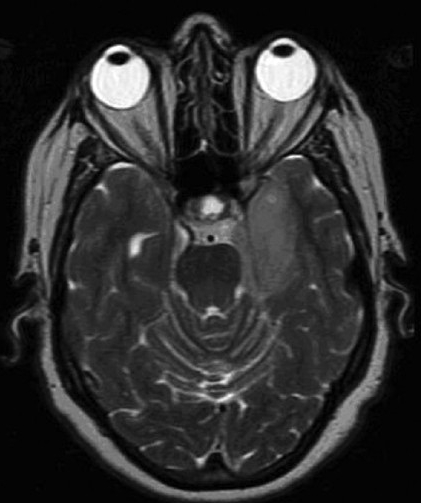
T2-weighted MR images without gadolinium. Left temporal lobe tumor with hyperintense signal

**Figure 2b:**
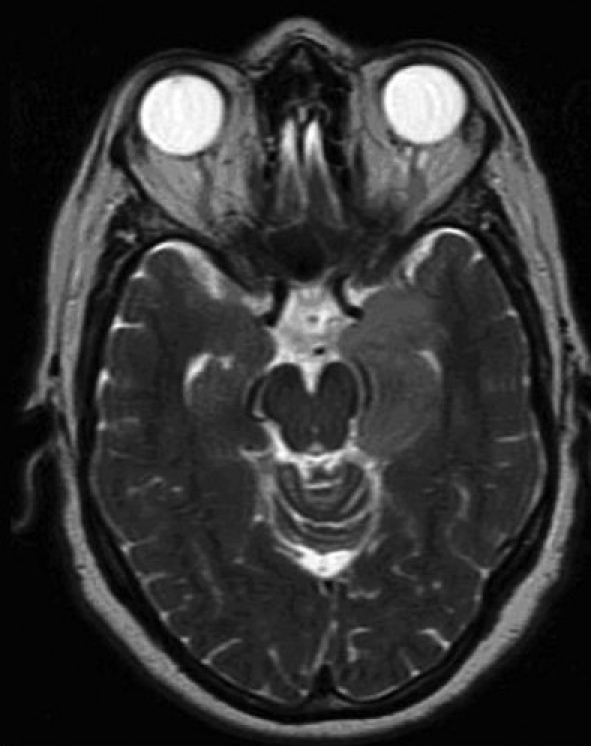
T2-weighted MR images without gadolinium. Left temporal lobe tumor with hyperintense signal

The patient was maintained on anti-convulsive medications. Subsequently, she underwent left-sided craniotomies for gross total resection of the left frontal tumor, anterior temporal lobectomy and subtotal resection of the temporal lobe tumor. Intra-operative microscope and BrainLab^®^, Munich, Germany, image-guided navigational system were utilized for the surgery. There was no attempt to remove or biopsy the right parietal tumor. The left frontal specimen showed primary high grade (WHO grade IV) primary neoplasm with astrocytic and neuronal differentiation [Figure 3a,b]. Immunohistochemical studies confirmed glial and neuronal differentiation of the tumor and excluded metastatic carcinoma. Interestingly, this high-grade frontal tumor showed also areas of lower cellularity with less mitotic activity, suggestive of a possible origin from an underlying low-grade glioma. On the other hand, the temporal tumor showed cerebral tissue with low-grade infiltrating astrocytic glioma (WHO grade II) only [Figure 4a,b].

**Figure 3a:**
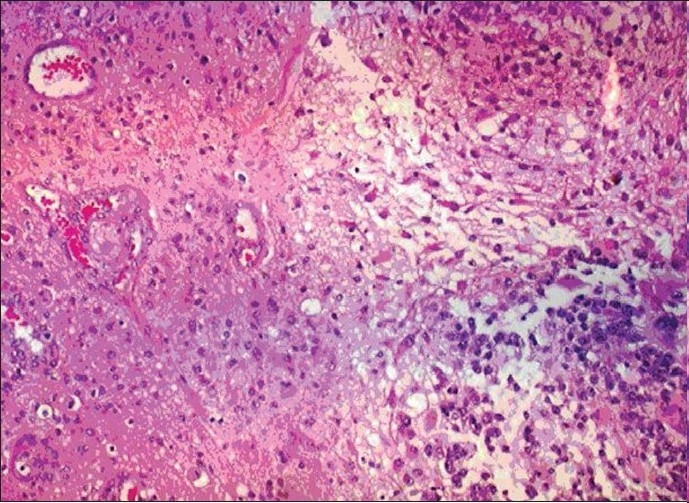
Photomicrograph of periphery of high-grade frontal tumor. Note cellular neoplasm on the right, and endothelial proliferation in the adjacent brain tissue, on the left. H and E stain, medium power magnification

**Figure 3b:**
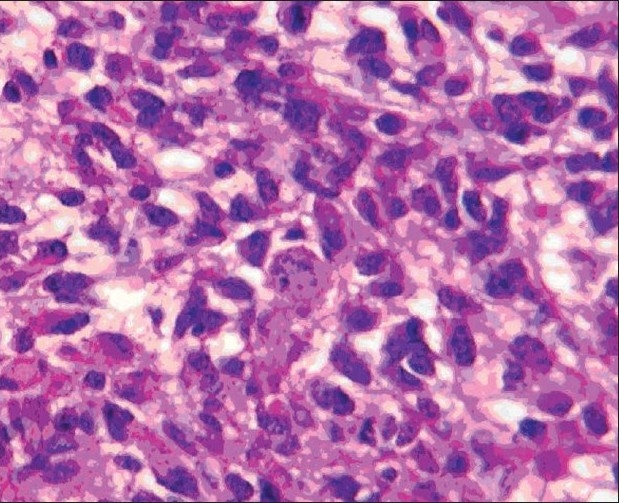
High-power magnification of the frontal tumor showing high cellularity, marked cellular atypia and atypical mitosis

**Figure 4a:**
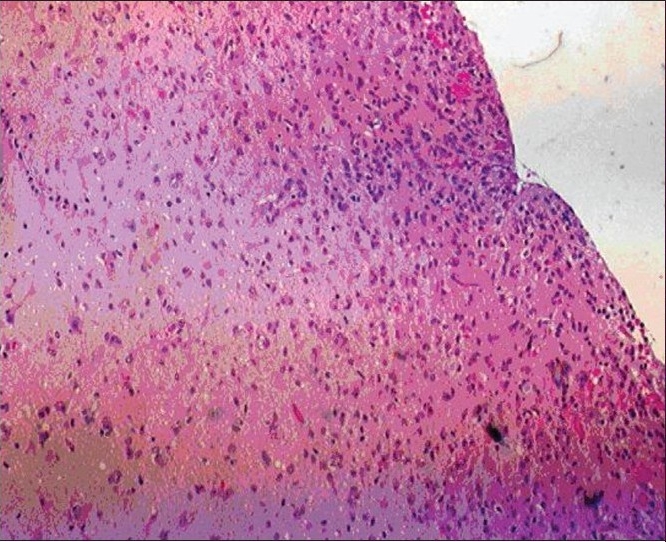
Low-power magnification of temporal lobe specimen showing low-grade astrocytoma infiltrating the cortex with subpial accumulation of tumor cells

**Figure 4b:**
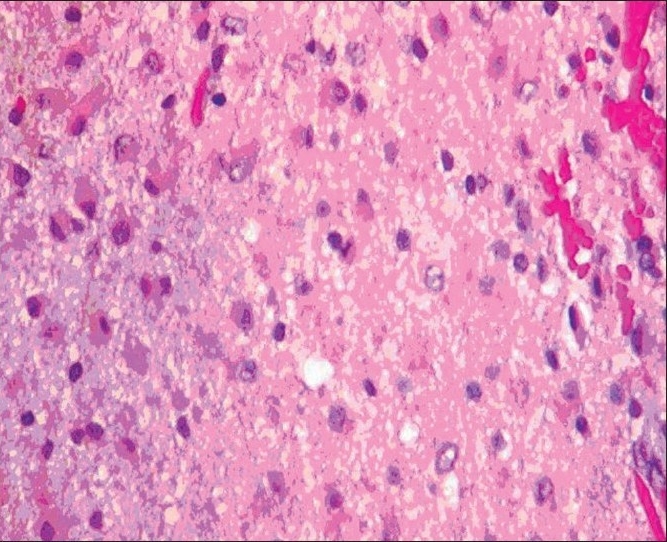
High-power magnification of temporal tumor showing astrocytic neoplasm with low cell density, mild nuclear atypia. No mitotic activity or endothelial proliferation seen

Postoperatively, the patient had an uneventful recovery except for a postoperative seizure, which was medically controlled. Despite early neurological response to adjuvent radiotherapy to the brain with a total dose of 40 Gy, the patient’s overall condition deteriorated a few months later secondary to the progression of her disease. She refused further treatment options and succumbed to her disease shortly thereafter. Autopsy was not performed.

## DISCUSSION

This patient presented with multiple synchronous primary brain tumors of distinctly different grades correlating radiographically and pathologically. The presence of two distinct low- and high-grade gliomas is a very rare condition with few reported cases occurring in different clinical contexts[13,8] which failed to demonstrate a clear MRI radiographical/pathological correlation as opposed to our case.

Gliomas can be generally divided into “circumscribed” grade I histologies versus “diffuse” or “infiltrating” higher grade II, III or IV varieties.[7] Conventional gadolinium-enhanced MR imaging is the standard technique for the evaluation of gliomas and typically demonstrates differences based on the grade of the tumor. In the absence of hemorrhage or dense calcification, the signal intensity of low-grade gliomas on T1-weighted images is usually homogeneous and comparable with or lower than that of gray matter. On T2-weighted images, gliomas typically are of high intensity, reflecting high water content in the tumor region. Many low-grade gliomas demonstrate little or no contrast enhancement, similar to the temporal lobe mass in this patient. The MR spectrum of more infiltrative, malignant and high-grade tumors overlaps those of lower grade lesions but generally the features are different.[20] Higher grade gliomas typically demonstrate heterogeneous tissue texture and signal intensity on TI-weighted images. This complex architecture reflects variable cellularity, as well as the common presence of necrosis, hemorrhage and cysts. On T2-weighted scans, the appearance of malignant gliomas is usually dominated by high signal intensity attributable to both the tumor tissue and reactive edema. Similar to our case in the lesions in the left frontal and right parietal lobes, glioblastomas commonly demonstrate irregular, thick-walled peripheral enhancement. The central nonenhancement, due to central necrosis, may reflect a combination of tumor outgrowing its blood supply as well as an intrinsic prothrombotic or vaso-occlusive event within the tumor.[4,1]

Three different pathways have been presented to characterize the pathophysiology of multifocal/multicentric high-grade gliomas.[17] In the first pathway a primary high-grade glioma may spread through cerebrospinal fluid or the white matter tracts to other locations. Usually when this occurs the primary lesion is clearly seen or may have been previously known. In the second pathway, multiple areas of high-grade glioma may arise *de novo*, without the presence of an underlying low-grade lesion. These tumors may arise from cells that, although not neoplastic in themselves, are nevertheless “primed” by an inherited or acquired genetic defect. In the third pathway, multiple areas of malignant degeneration from diffuse low-grade astrocytoma may occur. Occasionally, within a large area of brain infiltrated by a diffuse low-grade astrocytoma, multiple areas of malignant transformation occur, giving rise to multifocal high-grade glioma. This pathway may explain our patient’s findings in that the left frontal high-grade glioma showed mixed and low-grade features while the left temporal tumor remained entirely low grade in its histological features.

It is known that several mutations may contribute to the formation of secondary high-grade glioma which arise from preexisting low-grade gliomas. This type of tumor is relatively rare, has a female predominance and often has a characteristic mutation of TP53 and RB1, and increased expression of PDGFR.[16,6] Several other reports have demonstrated that multifocal gliomas are frequently associated with other primary malignancies including breast cancer or a family history of cancer in the context of Li-Fraumeni syndrome. It has been hypothesized that genetic alterations such as germline p53 mutations would account for this phenomenon.[10,12]

Multicentric and multifocal gliomas can be quite difficult to differentiate in most cases with the current available imaging technology and their histological features. True multicentric gliomas would have similar histological appearances. However, despite the heterogeneous nature of gliomas and the difference in their phenotype and grade, data suggest clonality in their origin.[3,9] The question remains whether multiple glial tumors originate from low-grade gliomas with subsequent degeneration of some foci supporting a monoclonal origin or these are separate tumors that coincidentally developed in the brain due to separate oncogenetic expression simultaneously, thus characterizing tumors of different cell lines.

## CONCLUSION

Although this case favors multicentric glioma arising from cell lines with varying degrees of differentiation suggestive of genetic predisposition or germline mutation, a correlation between the pathological findings and genetic features of the two distinct lesions would eventually be required to improve our understanding of this condition. Ultimately, identification of more genetic alterations resulting in the genesis, proliferation and invasiveness of malignant gliomas will better clarify the mechanism and direct future-targeted therapy in gliomas in general and more so in such atypical cases.
